# Swarm v3: towards tera-scale amplicon clustering

**DOI:** 10.1093/bioinformatics/btab493

**Published:** 2021-07-09

**Authors:** Frédéric Mahé, Lucas Czech, Alexandros Stamatakis, Christopher Quince, Colomban de Vargas, Micah Dunthorn, Torbjørn Rognes

**Affiliations:** UMR PHIM, CIRAD, Montpellier, France; PHIM Plant Health Institute, Univ Montpellier, CIRAD, INRAE, Institut Agro, IRD, Montpellier, France; Computational Molecular Evolution Group, Heidelberg Institute for Theoretical Studies, Heidelberg, Germany; Department of Plant Biology, Carnegie Institution for Science, Stanford, CA, USA; Computational Molecular Evolution Group, Heidelberg Institute for Theoretical Studies, Heidelberg, Germany; Institute of Theoretical Informatics, Karlsruhe Institute of Technology, Karlsruhe, Germany; Organisms and Ecosystems, Earlham Institute, Norwich, UK; Gut Microbes and Health, Quadram Institute, Norwich, UK; Warwick Medical School, University of Warwick, Coventry, UK; Sorbonne Université, CNRS, Station Biologique de Roscoff, UMR7144, ECOMAP, Roscoff, France; Research Federation for the study of Global Ocean Systems Ecology and Evolution, FR2022/Tara GOSEE, Paris, France; Natural History Museum, University of Oslo, Oslo, Norway; Eukaryotic Microbiology, University of Duisburg-Essen, Essen, Germany; Department of Informatics, University of Oslo, Oslo, Norway; Department of Microbiology, Oslo University Hospital, Rikshospitalet, Oslo, Norway

## Abstract

**Motivation:**

Previously we presented swarm, an open-source amplicon clustering programme that produces fine-scale molecular operational taxonomic units (OTUs) that are free of arbitrary global clustering thresholds. Here, we present swarm v3 to address issues of contemporary datasets that are growing towards tera-byte sizes.

**Results:**

When compared with previous swarm versions, swarm v3 has modernized C++ source code, reduced memory footprint by up to 50%, optimized CPU-usage and multithreading (more than 7 times faster with default parameters), and it has been extensively tested for its robustness and logic.

**Availability and implementation:**

Source code and binaries are available at https://github.com/torognes/swarm.

**Supplementary information:**

Supplementary data are available at *Bioinformatics* online.

## 1 Introduction 

In emerging planetary biology, large-scale amplicon sequencing datasets are used to unravel global ecological and evolutionary patterns within and across biomes and biota (de Vargas *et al.*, 2015; Mahé *et al.*, 2017; Giner *et al.*, 2020). With today’s sequencing platforms, such as Illumina and PacBio, single environmental diversity studies can produce massive amounts of data. A critical bioinformatics step in the handling of these massive metabarcoding datasets is to cluster the sequencing reads into operational taxonomic units (OTUs). OTUs are often used as units of comparison in downstream statistical analyses and are often interpreted as proxies for species and other taxa (Santoferrara *et al.*, 2020).

Swarm v1 (Mahé *et al.*, 2014) was introduced as a novel approach to cluster amplicons into OTUs, inspired by previous single-linkage methods, such as DOTUR (Schloss and Handelsman, 2005). The key underlying idea of swarm was to use a local, iterative, single-linkage clustering process to group closely related sequences (by default with one difference in their nucleotide sequences, i.e. *d *=* *1). Swarm’s clustering process differs from global clustering threshold approaches that apply an arbitrary fixed minimal similarity between the OTU seed and other OTU members; often set at 97% or 98% (Edgar *et al.*, 2010), or from model-based noise-filtering methods, such as DADA2 (Callahan *et al.*, 2016) and Deblur (Amir *et al.*, 2017). The recommended usage of these methods is to process samples or sequencing runs independently and then to merge the results. Swarm offers a fast alternative allowing users to (re-)process entire datasets at once. Swarm v2 (Mahé *et al.*, 2015) implemented in C++ two additional features to refine clustering: OTU-breaking that splits OTUs that are only linked via low-abundant sequences (--no-otu-breaking to disable); and the merging that grafts low-abundant OTUs onto higher-abundant OTUs (--fastidious to enable).

Swarm v2 was completely implemented in C++ and was substantially faster due to algorithmic advances when used with default parameters (*d *=* *1). There was still room for improvement. There were issues with code standardization that could limit compile-time optimization and raise warnings or errors with future compilers (Darriba *et al.*, 2018; Wilson *et al.*, 2014). The code could only be executed on GNU/Linux and macOS on x86-64 CPUs. And although swarm v2 was multithreaded and fast, its time and memory requirements could become a limiting factor on very large current and future datasets, especially as amplicon sequences become longer. Swarm v3 addresses these issues.

## 2 Code quality and portability

Following the recommendations of Darriba *et al.* (2018), swarm v3 features a substantially revised and improved documentation (e.g. help and man page), as well as clearer and more helpful warnings and error messages. Swarm’s logic and behavior have been tested extensively via automatically generated input (afl-fuzz; https://lcamtuf.coredump.cx/afl/) and 669 hand-crafted functional software tests (https://github.com/frederic-mahe/swarm-tests/), covering more than 95% of swarm’s code (the remaining code is CPU architecture-specific). The Codecov (https://codecov.io) tool tracks code coverage evolution, and the Travis-CI (https://travis-ci.org) suite automatically executes the test suite on each new code modification to prevent regressions.

To facilitate swarm’s long-term maintenance and portability, advanced compiler options [gcc (https://gcc.gnu.org) and clang (https://clang.llvm.org)] as well as state-of-the-art static [cppcheck (http://cppcheck.sourceforge.net) and clang-tidy (https://clang.llvm.org/extra/clang-tidy/)] and dynamic C++ analyzers [valgrind (https://www.valgrind.org)] were used to detect unsafe or deprecated code not reported by commonly used compiler options. More than 1600 warnings were fixed so far, improving swarm’s global code quality score as assessed by SoftWipe (Zapletal *et al.*, 2020) from 5.2 to 6.6 out of 10. Swarm has now been ported to new combinations of CPU architectures and operating systems: Microsoft’s Windows on x86-64, GNU/Linux and macOS on ARM 64 and GNU/Linux on POWER8, in addition to the already available versions for GNU/Linux and macOS on x86-64.

## 3 Time and space optimization, real-world results

DNA sequences are stored *in silico* as strings of the four characters A, C, G and T. Rather than using a byte of memory for storing each nucleotide, it is possible to only use two bits. Thereby, four nucleotides can be stored per byte. This compression reduces the global memory-footprint but also requires some storage overhead and additional encoding-decoding operations as CPUs cannot operate directly on anything smaller than a byte. To alleviate this, swarm v3 deploys a faster hash function (Zobrist, 1970) and an efficient Bloom filter (Putze *et al.*, 2009), and was re-written to operate on fixed-length chunks of compressed sequences, rather than on individual nucleotides (see Supplementary File). It should be noted that this new algorithm only applies to the default value for swarm’s *d* parameter (*d *=* *1). Higher *d* values use the same algorithm as in swarm v2.

On a dataset of 10.6 million unique SSU-rRNA V4 sequences (representing 31.6 million reads, 380 bp on average, Mahé *et al.*, 2017), and a series of subsamplings (1% and 10–90% steps), swarm v3 outperformed swarm v2 in every performance metric, while yielding exactly identical clustering results. With both versions running on 1 core, v3 was more than 7 times faster than v2. When both were running on 16 cores, v3 was about 10 times faster than v2. The memory requirement of v3 was about half that of v2 (Supplementary Fig. S1). Comparable results were obtained on a second dataset of 10.6 million unique SSU-rRNA V9 sequences (130 bp on average, de Vargas *et al.*, 2015), but with a less pronounced memory-footprint reduction as the storage overhead of two-bit compressed sequences has a larger impact with shorter sequences (see Supplementary Figs. S2, S3 and Supplementary File for a detailed benchmark description).

When using the merging option (named fastidious), swarm v3 is more than 5 times faster for SSU-rRNA V9 (130 bp), and more than 9 times faster for SSU-rRNA V4 (380 bp) (Supplementary Fig. S2). The memory-footprint is only reduced by 5–10% due to the fact that the fastidious algorithm relies on a Bloom filter to store hash values instead of DNA sequences, and therefore does not profit from the two-bit sequence compression.

## 4 Conclusion

Swarm v3 is a clustering method designed to maximize taxonomic resolution, sensitivity and speed. If coupled with ‘lossy’ post-clustering filtering steps, such as chimera detection, quality filtering and multi-sample co-occurrence patterns (e.g. Frøslev *et al.*, 2017), swarm has the potential to yield robust, single-nucleotide resolution results. Swarm v3 can be used on short and long read metabarcoding data (with sequences up to 10 Mbp when using *d *=* *1), or on meta-transcriptomic/genomic data that has been subsampled from the same locus. It offers a comprehensive set of options that gives users full-control and access to intermediate internal data, such as the complete pairwise sequence network (see Forster *et al.*, 2020, for a usage example). Swarm v3 is open-source, actively maintained, portable and efficient, thus reducing the need for expensive computational resources. As an example, the UniEuk project (Berney *et al.*, 2017) gathered from the global research community an SSU-rRNA V4 dataset with nearly 324 million unique sequences (123 billion nucleotides), more than three times the volume of the recently published Earth Microbiome Project (Thompson *et al.*, 2017). Using default parameters, swarm v3 required 50 min to cluster the UniEuk dataset on a 16-core system. We estimate that it would take less than six hours on the same machine to process a one trillion nucleotide, or one tera-byte dataset.

## Supplementary Material

btab493_Supplementary_Data
