# Supplementary material for: Swarm v3: towards tera-scale amplicon clustering
Source: Bioinformatics. 2021 Jul 9;38(1):267–9. doi: 10.1093/bioinformatics/btab493 (PMC8696092; doi:10.1093/bioinformatics/btab493)
Supplement: btab493_Supplementary_Data [file btab493_supplementary_data.pdf]

Swarm v3: towards tera-scale sequence clustering

Table of Contents

- 1. Disclaimer
- 2. Methods
- 3. Benchmark
- 4. Plot creation
- 5. Supplementary Figures
  - 5.1. Supp. Fig. 1: Performance improvement of swarm version 3 relative to version 2
  - 5.2. Supp. Fig. 2: Detailed speed of swarm version 3 relative to version 2
  - 5.3. Supp. Fig. 3: Detailed memory footprint requirements of swarm version 3 relative to version 2
- 6. References

Frédéric Mahé, Lucas Czech, Alexandros Stamatakis, Christopher Quince, Colomban de Vargas, Micah Dunthorn, and Torbjørn Rognes

1 Disclaimer

The purpose of this document is to provide the reader with details on the bioinformatics methods used to prepare this paper. The code snippets and shell commands presented here were executed on a CentOS Linux release 7.5, and might have to be adapted to your particular system. Use them carefully.

2 Methods

Swarm 3.0 introduces a new hash function to identify similar sequences. Swarm previously used the Google CityHash library (https://github.com/google/cityhash) to perform hashing of sequences, but we have now switched to a simpler hash function using a tabulated hashing approach (Zobrist, 1970). The hash function is initialized with a set of 4n pseudo-random 64-bit constants representing any of the four nucleotides in any of the n positions in the longest input sequence. These constants are then used to compute the hash value of each sequence via a bit-wise XOR. This results in a fast hash function that can be updated incrementally, and has been mathematically proved to be of high quality (3-independent and universal). During an incremental update, small changes in the sequence (e.g., a single base substitution) require just a few operations on the hash value (e.g., two XOR operations) instead of a full recalculation. Swarm generates all the so-called microvariants of each sequence in order to find all other sequences with distance 1 that should be linked during clustering. Each microvariant of a sequence involves only either one single base substitution, single base insertion or single base deletion. When generating microvariants, swarm now avoids generating the full variant sequences, and only generates the hash of each variant, which can easily be performed in an incremental manner. This results in a substantial speed improvement over swarm version 2. All input sequences were initially hashed and stored in a hash table. In swarm version 3, sequences are now additionally recorded in an efficient Bloom filter (Putze et al., 2009), allowing swarm to quickly reject requests for sequences that are not present.

3 Benchmark

The "18S V4 Neotropical Soils" and "18S V9 TARA OCEANS" fasta files are available on demand (1.5 GB of gziped data in total).

Computational experiments were performed on the IFB cluster (France), using DELL C6320 nodes with 2x Intel Xeon E5-2695v3 (2.3GHz, 14 cores), 256GB RAM, and CentOS Linux release 7.5.1804 (see the cluster description).

To ensure comparability, each swarm 3 vs swarm 2 comparison was executed sequentially on the same node. Swarm being a deterministic algorithm, it normally shows very little variance in execution times when running in a single-user environment (typically less than 2% of variance). However, in multi-user environments such as a busy cluster, CPU and I/O load can vary significantly and affect runtime comparisons. To mitigate that we performed 30 repeats per setting and excluded outliers (see R code in the next section).

```
cd ${HOME}

## set up
mkdir -p swarm3_benchmark/{data,results,src}
cd ./swarm3_benchmark/
```

```
## produce the subsampled files
(cd ./data/
 SSU_V9="18S_V9_496_samples_100.fas"
 SSU_V4="18S_V4_175_samples_10M_100.fas"
 module purge && module load gcc/9.3.0
 export LC_ALL=C
 VSEARCH="${HOME}/src/vsearch/bin/vsearch"

 for FASTA_INPUT in "${SSU_V4}" "${SSU_V9}" ; do
     for PERCENTAGE in 1 10 20 30 40 50 60 70 80 90 ; do
         "${VSEARCH}" \
             --fastx_subsample "${FASTA_INPUT}" \
             --randseed 1 \
             --sample_pct ${PERCENTAGE} \
             --sizein \
             --sizeout \
             --fastaout "${FASTA_INPUT/_100.fas}_${PERCENTAGE}.fas"
     done
 done
md5sum -c MD5SUM
)


## create swarm binaries
(cd ./src/
 module purge && module load gcc/9.3.0
 git clone https://github.com/torognes/swarm.git
 cd swarm/src/
 make -j
 mv ../bin/swarm ../../swarm3
 make clean
 git checkout tags/v2.2.2
 make -j
 mv ../bin/swarm ../../swarm2
 make clean
 git checkout master
 cd ../..
 ./swarm2 -v
 ./swarm3 -v
 rm -rf ./swarm/
)


## swarm clustering (for each condition, swarm 2 and 3 run
## sequentially, allowing for direct comparison)
(cd ./src/
 GLOBAL_MEMORY=72
 for FLAVOR in normal fastidious ; do
     for FASTA_FILE in $(ls ../data/*.fas | sort -V) ; do
         NUMBER_OF_SEQUENCES=$(grep -c "^>" "${FASTA_FILE}")
         NUMBER_OF_NUCLEOTIDES=$(grep -v "^>" "${FASTA_FILE}" | tr -d "\n" | wc -c)
         for THREADS in 16 8 4 1 ; do
             for ITERATION in {1..30} ; do
                 sbatch \
                     --cpus-per-task="${THREADS}" \
                     --mem=${GLOBAL_MEMORY}G \
                     ./swarm.sh \
                     "${FASTA_FILE}" \
                     "${THREADS}" \
                     "${ITERATION}" \
                     "${NUMBER_OF_SEQUENCES}" \
                     "${NUMBER_OF_NUCLEOTIDES}" \
                     "${FLAVOR}"
             done
         done
     done
 done
)
```

4 Plot creation

Outlier values for the different metrics and for each set of 30 repeats were detected and excluded following Tukey's method (Tukey 1977, pp. 43-44). Points represent average values, and error-bars represent 95%-confidence intervals.

```r
## R version 3.5.2 (2018-12-20) -- "Eggshell Igloo"

library(tidyverse)
library(RColorBrewer)
library(scales)

setwd("${HOME}/swarm3_benchmark/results/")
input <- "all.table"
mycolumns <- c("method", "flavor", "dataset", "percentage",
               "sequences", "nucleotides", "threads", "iteration",
               "speed", "memory", "CPU")

## detect outliers (Tukey method, not influenced by extreme values)
## outlier on the upper side = 3rd quartile + 1.5 * IQR
## outlier on the lower side = 1st quartile – 1.5 * IQR
## IQR (interquartile range) = 3rd quartile – 1st quartile
is_outlier <- function(x) {
    return(x < quantile(x, 0.25) - 1.5 * IQR(x) | x > quantile(x, 0.75) + 1.5 * IQR(x))
}


## load the data
read_tsv(input, col_names = mycolumns) %>%
    select(-CPU) %>%
    mutate(threads = as.factor(threads)) %>%
    mutate(flavor = if_else(flavor == "normal", "default", "fastidious")) %>%
    mutate(marker = if_else(grepl("^18S_V4", dataset), "18S rRNA V4", "18S rRNA V9")) %>%
    gather("metric", "value", speed, memory) %>%
    spread(method, value) %>%
    mutate(ratio = if_else(metric == "speed", swarm2 / swarm3, swarm3 / swarm2)) %>%
    select(-swarm2, -swarm3) %>%
    group_by(marker, flavor, sequences, threads, metric) %>%
    mutate(outlier = is_outlier(ratio),
           ratio_mean = mean(ratio),
           ratio_conf_int = 1.96 * sd(ratio) / sqrt(length(ratio))) %>%
    filter(outlier == FALSE) %>%
    ungroup() -> d


## number of thread levels and color palette
n_blues_levels <- brewer.pal.info["Blues",]$maxcolor
n_thread_levels <- d %>% pull(threads) %>% nlevels()
n <- n_blues_levels - n_thread_levels
thread_colors <- brewer.pal(n_blues_levels, "Blues")[-(1:n)]
thread_colors <- c("#4292C6", "#2171B5", "#08519C", "#000000")  # replace darkest shade with black

## normality tests (Shapiro-Wilk, very sensitive to deviation, n = 40)
d %>%
    filter(metric == "speed") %>%
    group_by(marker, flavor, sequences, threads) %>%
    mutate(p_values = shapiro.test(ratio)$p.value) %>%
    ungroup() %>%
    filter(p_values <= 0.01) %>%
    count(marker, flavor, sequences, threads)
## some conditions are still strictly deviating from a normal
## distribution, even after removing outliers


## ---------------------------------------------------------------- summary plot

## speed, memory footprint for 18SV4 (default parameters)
d %>%
    filter(marker == "18S rRNA V4" & flavor == "default") %>%
    mutate(metric = case_when(
               metric == "memory" ~ "memory footprint",
               TRUE ~ metric)) %>%
    mutate(metric = fct_rev(metric)) %>%
    ggplot(aes(x = sequences / 10**6,
               y = ratio_mean,
               group = threads,
               colour = threads)) +
    geom_line() +
    geom_pointrange(aes(ymin = ratio_mean - ratio_conf_int,
                        ymax = ratio_mean + ratio_conf_int),
                    position = position_dodge(width = 0.4)) +
    facet_grid(metric ~ ., scales = "free_y") +
```

```r
    expand_limits(x = 0, y = c(0, 0.6)) +
    scale_y_continuous(name = "swarm 3 vs. swarm 2 (performance ratios)") +
    scale_x_continuous(name = "number of sequences (in millions)") +
    theme_bw(base_size = 16) +
    theme(legend.justification = c(0, 0),
          legend.position = c(0.725, 0.02),
          legend.background = element_rect(colour = "gray90", size = 1),
          legend.text = element_text(size = 10),
          legend.title = element_text(size = 12)) +
    scale_colour_manual(values = thread_colors,
                        guide = guide_legend(reverse = TRUE))

ggsave("figureS1_18SV4_swarm_default_all_metrics.png",
       width = 4.5, height = 5.5, units = "in", dpi = "print")


## ------------------------------------------------------------ detailled plots

## speed plot
d %>%
    filter(metric == "speed") %>%
    ggplot(aes(x = sequences / 10**6,
               y = ratio_mean,
               group = threads,
               colour = threads)) +
    geom_line() +
    geom_pointrange(aes(ymin = ratio_mean - ratio_conf_int,
                        ymax = ratio_mean + ratio_conf_int),
                    position = position_dodge(width = 0.4)) +
    facet_grid(flavor ~ marker) +
    expand_limits(x = 0, y = 0) +
    scale_y_continuous(name = "swarm 3's speed\n(times faster than swarm 2)") +
    scale_x_continuous(name = "number of sequences (in millions)") +
    theme_bw(base_size = 16) +
    theme(legend.justification = c(0, 0),
          legend.position = c(0.01, 0.01),
          legend.background = element_rect(colour = "gray90", size = 1),
          legend.text = element_text(size = 10),
          legend.title = element_text(size = 12)) +
    scale_colour_manual(values = thread_colors,
                        guide = guide_legend(reverse = TRUE))

ggsave("figureS2_speedup.png", dpi = "print")


## memory footprint plot (no visible dependency to thread number)
d %>%
    filter(metric == "memory") %>%
    ggplot(aes(x = sequences / 10**6,
               y = ratio_mean,
               group = threads,
               colour = threads)) +
    geom_line() +
    geom_pointrange(aes(ymin = ratio_mean - ratio_conf_int,
                        ymax = ratio_mean + ratio_conf_int),
                    position = position_dodge(width = 0.4)) +
    facet_grid(flavor ~ marker) +
    coord_cartesian(ylim = c(0, 1)) +
    expand_limits(x = 0) +
    scale_y_continuous(name = "swarm 3's memory footprint\n(compared with swarm 2)") +
    scale_x_continuous(name = "number of sequences (in millions)") +
    theme_bw(base_size = 16) +
    theme(legend.justification = c(0, 0),
          legend.position = c(0.01, 0.01),
          legend.background = element_rect(colour = "gray90", size = 1),
          legend.text = element_text(size = 10),
          legend.title = element_text(size = 12)) +
    scale_colour_manual(values = thread_colors,
                        guide = guide_legend(reverse = TRUE))

ggsave("figureS3_memory_footprint.png", dpi = "print")

quit(save = "no")
```

5 Supplementary Figures

5.1 Supp. Fig. 1: Performance improvement of swarm version 3 relative to version 2

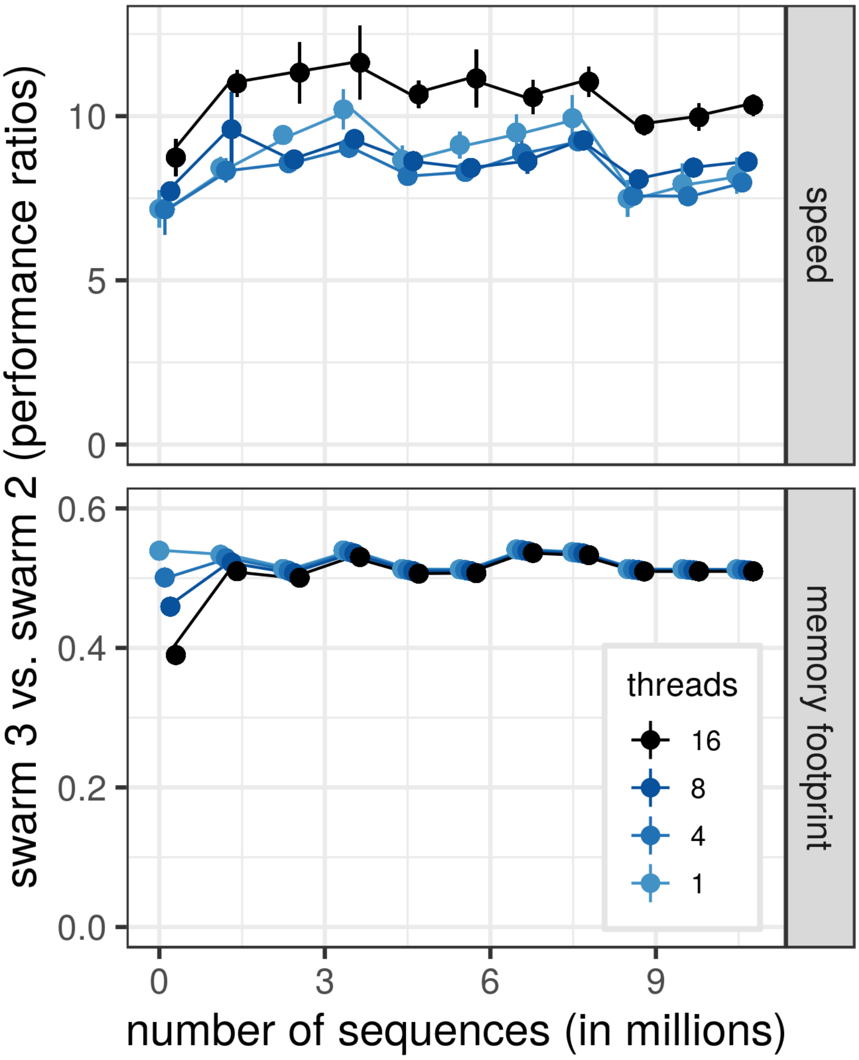

The speed (top) and memory footprint (bottom) of swarm v3 relative to swarm v2 is shown. The relative performance is indicated on the vertical axis, while the number of sequences is indicated on the horizontal axis. Performance was assessed on a dataset of 10.6 million unique SSU-rRNA V4 sequences with an average length of 380 bp. Analyses were performed by subsampling at 1% and at 10% intervals from 10% to 90% as well as on the full dataset. The experiments were run on 1, 4, 8 or 16 cores as indicated by the light blue to dark blue colour, 30 repeats per condition. The fastidious option was disabled. Vertical bars represent 95%-confidence intervals.

5.2 Supp. Fig. 2: Detailed speed of swarm version 3 relative to version 2

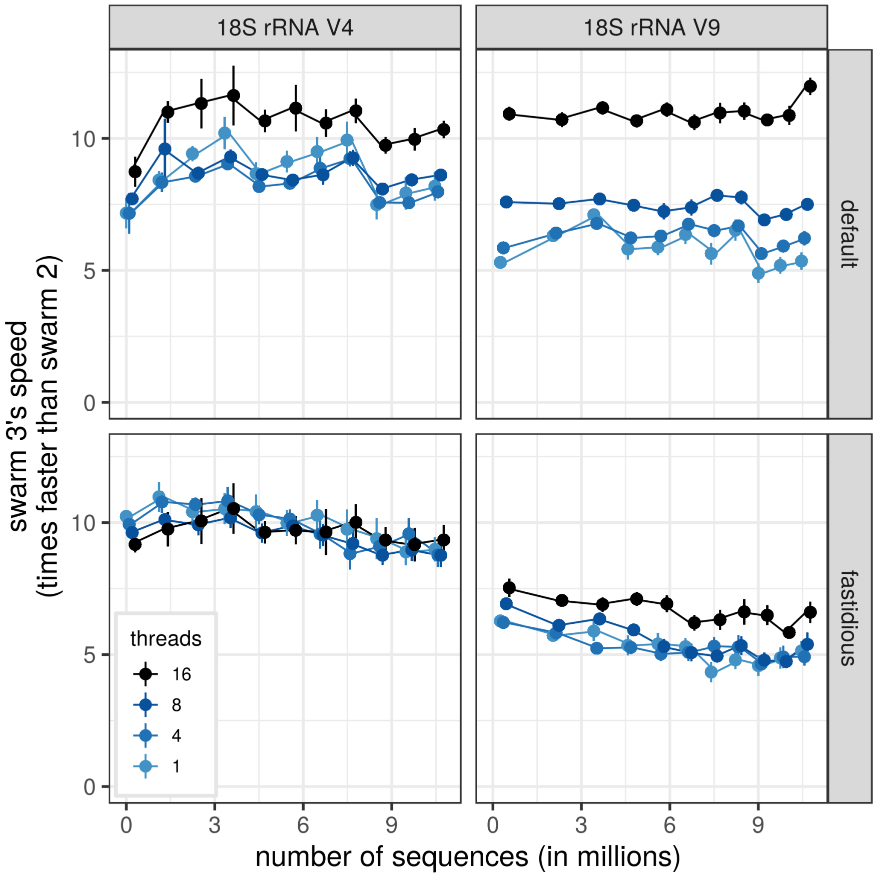

The four panels show the speed of swarm v3 relative to swarm v2 using the default non-fastidious clustering mode (top) and the optional fastidious clustering mode (bottom) on SSU-rRNA V4 sequences (left) and the shorter V9 sequences (right). The relative performance is indicated on the vertical axis, while the number of sequences is indicated on the horizontal axis. Performance was assessed using a dataset of 10.6 million unique SSU-rRNA V4 sequences with an average length of 380 bp (left), and on a dataset of 10.6 million unique SSU-rRNA V9 sequences with an average length of 130 bp (right). Analyses were performed by subsampling at 1% and at 10% intervals from 10% to 90% as well as on the full datasets. The experiments were run on 1, 4, 8 or 16 cores as indicated by the light blue to dark blue colour, 30 repeats per condition. Vertical bars represent 95%-confidence intervals.

5.3 Supp. Fig. 3: Detailed memory footprint requirements of swarm version 3 relative to version 2

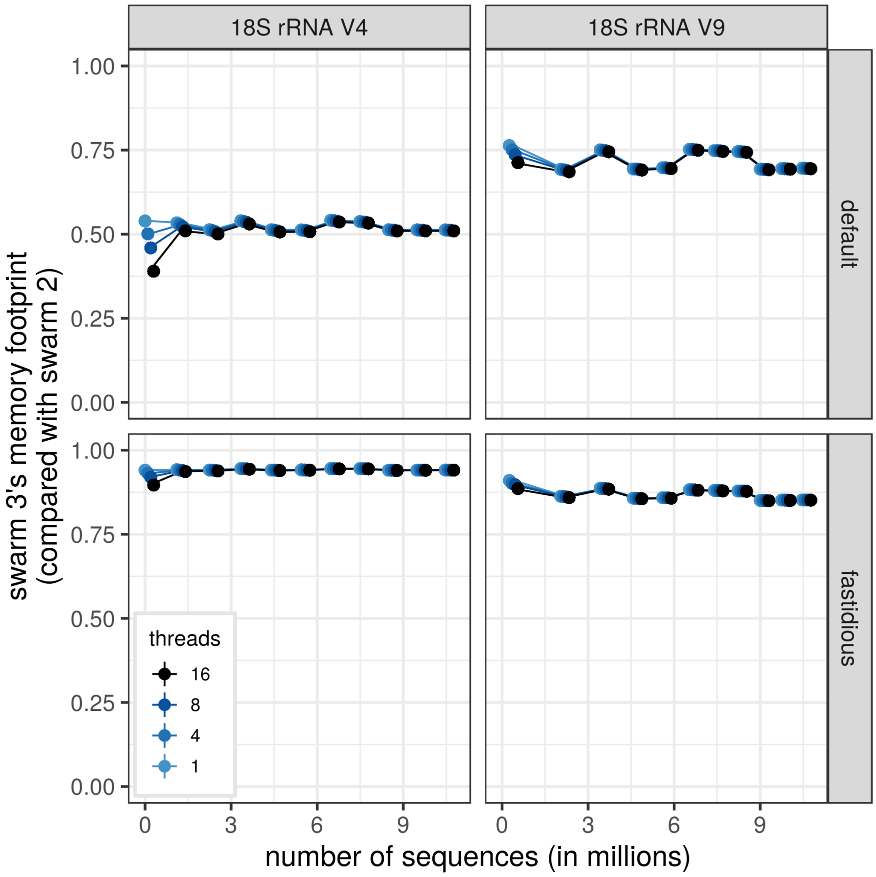

The four panels show the memory footprint of swarm v3 relative to swarm v2 using the default non-fastidious clustering mode (top) and the optional fastidious clustering mode (bottom) on SSU-rRNA V4 sequences (left) and the shorter V9 sequences (right). The relative memory usage is indicated on the vertical axis, while the number of sequences is indicated on the horizontal axis. The performance was analysed using a dataset of 10.6 million unique SSU-rRNA V4 sequences with an average length of 380 bp (left), and on a dataset of 10.6 million unique SSU-rRNA V9 sequences with an average length of 130 bp (right). Analyses were performed by subsampling at 1% and at 10% intervals from 10% to 90% as well as on the full dataset. The experiments were run with 1, 4, 8 or 16 cores as indicated by the light blue to dark blue colour, 30 repeats per condition. Vertical bars represent 95%-confidence intervals.

6 References

Mahé F, Rognes T, Quince C, de Vargas C, Dunthorn M. 2015. Swarm v2: highly-scalable and high-resolution amplicon clustering. PeerJ 3:e1420 https://doi.org/10.7717/peerj.1420

Putze F, Sanders P, Singler J (2009) Cache-, Hash- and Space-Efficient Bloom Filters. Journal of Experimental Algorithmics, 14, 4. https://doi.org/10.1145/1498698.1594230

R Core Team (2018). R: A language and environment for statistical computing. R Foundation for Statistical Computing, Vienna, Austria. URL https://www.r-project.org/.

Rognes T, Flouri T, Nichols B, Quince C, Mahé F. 2016. VSEARCH: a versatile open source tool for metagenomics. PeerJ 4:e2584 https://doi.org/10.7717/peerj.2584

Tukey, John Wilder (1977) Exploratory Data Analysis. Addison-Wesley. ISBN 978-0-201-07616-5.

Wickham et al., (2019). Welcome to the tidyverse. Journal of Open Source Software, 4(43), 1686, https://doi.org/10.21105/joss.01686

Zobrist AL (1970) A New Hashing Method with Application for Game Playing. Tech. Rep. 88, Computer Sciences Department, University of Wisconsin, Madison, Wisconsin, USA. http://digital.library.wisc.edu/1793/57624

Date: 2021-06-30 mer. 00:00
Author: Frédéric Mahé
Created: 2021-06-30 mer. 12:02
Validate
